# Supplementary material for: Unveiling the Bioactive Potential of the Invasive Jellyfish Phyllorhiza punctata Through Integrative Transcriptomic and Proteomic Analyses
Source: Biomolecules. 2025 Aug 4;15(8):1121. doi: 10.3390/biom15081121 (PMC12383608; doi:10.3390/biom15081121)
Supplement: Supplementary file 1 [file biomolecules-15-01121-s001.zip › Suplementary Material/FileS3/PEPFOLD4/XP_065053058_5367/clusters.html]

 PEP-FOLD clusters


Download archive of all models  here   

|  |  |  |
| --- | --- | --- |
| # | file | sOPEP |
| # cluster 1 - Size: 1 |  |  |
|  | XP\_065053058\_5367-model1 | -37.3999 |
| # cluster 2 - Size: 3 |  |  |
|  | XP\_065053058\_5367-model2 | -30.8138 |
|  | XP\_065053058\_5367-model2.1 | -23.5123 |
|  | XP\_065053058\_5367-model2.2 | -21.4663 |
| # cluster 3 - Size: 1 |  |  |
|  | XP\_065053058\_5367-model3 | -28.7986 |
| # cluster 4 - Size: 1 |  |  |
|  | XP\_065053058\_5367-model4 | -26.5126 |
| # cluster 5 - Size: 1 |  |  |
|  | XP\_065053058\_5367-model5 | -25.9382 |
| # cluster 6 - Size: 1 |  |  |
|  | XP\_065053058\_5367-model6 | -25.6334 |
| # cluster 7 - Size: 1 |  |  |
|  | XP\_065053058\_5367-model7 | -23.6151 |
| # cluster 8 - Size: 1 |  |  |
|  | XP\_065053058\_5367-model8 | -23.4258 |
| # cluster 9 - Size: 1 |  |  |
|  | XP\_065053058\_5367-model9 | -22.9761 |
| # cluster 10 - Size: 1 |  |  |
|  | XP\_065053058\_5367-model10 | -22.583 |
| # cluster 11 - Size: 4 |  |  |
|  | XP\_065053058\_5367-model11 | -22.5547 |
|  | XP\_065053058\_5367-model11.1 | -18.745 |
|  | XP\_065053058\_5367-model11.2 | -18.0043 |
|  | XP\_065053058\_5367-model11.3 | -16.7496 |
| # cluster 12 - Size: 2 |  |  |
|  | XP\_065053058\_5367-model12 | -21.7958 |
|  | XP\_065053058\_5367-model12.1 | -21.7107 |
| # cluster 13 - Size: 17 |  |  |
|  | XP\_065053058\_5367-model13 | -20.8906 |
|  | XP\_065053058\_5367-model13.1 | -20.6617 |
|  | XP\_065053058\_5367-model13.2 | -20.4249 |
|  | XP\_065053058\_5367-model13.3 | -19.8402 |
|  | XP\_065053058\_5367-model13.4 | -19.8386 |
|  | XP\_065053058\_5367-model13.5 | -19.6993 |
|  | XP\_065053058\_5367-model13.6 | -19.4576 |
|  | XP\_065053058\_5367-model13.7 | -19.3103 |
|  | XP\_065053058\_5367-model13.8 | -19.1828 |
|  | XP\_065053058\_5367-model13.9 | -19.0712 |
|  | XP\_065053058\_5367-model13.10 | -18.8677 |
|  | XP\_065053058\_5367-model13.11 | -18.7657 |
|  | XP\_065053058\_5367-model13.12 | -18.6428 |
|  | XP\_065053058\_5367-model13.13 | -18.4345 |
|  | XP\_065053058\_5367-model13.14 | -17.6063 |
|  | XP\_065053058\_5367-model13.15 | -17.406 |
|  | XP\_065053058\_5367-model13.16 | -15.8303 |
| # cluster 14 - Size: 1 |  |  |
|  | XP\_065053058\_5367-model14 | -20.8363 |
| # cluster 15 - Size: 2 |  |  |
|  | XP\_065053058\_5367-model15 | -20.7901 |
|  | XP\_065053058\_5367-model15.1 | -19.8222 |
| # cluster 16 - Size: 3 |  |  |
|  | XP\_065053058\_5367-model16 | -20.3154 |
|  | XP\_065053058\_5367-model16.1 | -16.2052 |
|  | XP\_065053058\_5367-model16.2 | -15.02 |
| # cluster 17 - Size: 2 |  |  |
|  | XP\_065053058\_5367-model17 | -20.2482 |
|  | XP\_065053058\_5367-model17.1 | -14.0755 |
| # cluster 18 - Size: 2 |  |  |
|  | XP\_065053058\_5367-model18 | -19.9378 |
|  | XP\_065053058\_5367-model18.1 | -18.441 |
| # cluster 19 - Size: 1 |  |  |
|  | XP\_065053058\_5367-model19 | -19.9031 |
| # cluster 20 - Size: 1 |  |  |
|  | XP\_065053058\_5367-model20 | -19.8598 |
| # cluster 21 - Size: 1 |  |  |
|  | XP\_065053058\_5367-model21 | -19.636 |
| # cluster 22 - Size: 1 |  |  |
|  | XP\_065053058\_5367-model22 | -19.6329 |
| # cluster 23 - Size: 1 |  |  |
|  | XP\_065053058\_5367-model23 | -19.5048 |
| # cluster 24 - Size: 1 |  |  |
|  | XP\_065053058\_5367-model24 | -19.4464 |
| # cluster 25 - Size: 1 |  |  |
|  | XP\_065053058\_5367-model25 | -19.382 |
| # cluster 26 - Size: 1 |  |  |
|  | XP\_065053058\_5367-model26 | -19.3334 |
| # cluster 27 - Size: 1 |  |  |
|  | XP\_065053058\_5367-model27 | -19.3208 |
| # cluster 28 - Size: 5 |  |  |
|  | XP\_065053058\_5367-model28 | -19.2176 |
|  | XP\_065053058\_5367-model28.1 | -18.9703 |
|  | XP\_065053058\_5367-model28.2 | -18.884 |
|  | XP\_065053058\_5367-model28.3 | -17.8229 |
|  | XP\_065053058\_5367-model28.4 | -17.0114 |
| # cluster 29 - Size: 2 |  |  |
|  | XP\_065053058\_5367-model29 | -19.1737 |
|  | XP\_065053058\_5367-model29.1 | -18.224 |
| # cluster 30 - Size: 7 |  |  |
|  | XP\_065053058\_5367-model30 | -19.068 |
|  | XP\_065053058\_5367-model30.1 | -19.0294 |
|  | XP\_065053058\_5367-model30.2 | -18.9754 |
|  | XP\_065053058\_5367-model30.3 | -18.9512 |
|  | XP\_065053058\_5367-model30.4 | -17.3353 |
|  | XP\_065053058\_5367-model30.5 | -16.5495 |
|  | XP\_065053058\_5367-model30.6 | -16.1793 |
| # cluster 31 - Size: 1 |  |  |
|  | XP\_065053058\_5367-model31 | -19.0335 |
| # cluster 32 - Size: 2 |  |  |
|  | XP\_065053058\_5367-model32 | -18.7335 |
|  | XP\_065053058\_5367-model32.1 | -18.4612 |
| # cluster 33 - Size: 1 |  |  |
|  | XP\_065053058\_5367-model33 | -18.6895 |
| # cluster 34 - Size: 1 |  |  |
|  | XP\_065053058\_5367-model34 | -18.6788 |
| # cluster 35 - Size: 1 |  |  |
|  | XP\_065053058\_5367-model35 | -18.6452 |
| # cluster 36 - Size: 1 |  |  |
|  | XP\_065053058\_5367-model36 | -17.7531 |
| # cluster 37 - Size: 1 |  |  |
|  | XP\_065053058\_5367-model37 | -17.2932 |
| # cluster 38 - Size: 1 |  |  |
|  | XP\_065053058\_5367-model38 | -16.5433 |
| # cluster 39 - Size: 5 |  |  |
|  | XP\_065053058\_5367-model39 | -16.4128 |
|  | XP\_065053058\_5367-model39.1 | -16.277 |
|  | XP\_065053058\_5367-model39.2 | -16.2292 |
|  | XP\_065053058\_5367-model39.3 | -15.6396 |
|  | XP\_065053058\_5367-model39.4 | -14.9853 |
| # cluster 40 - Size: 3 |  |  |
|  | XP\_065053058\_5367-model40 | -16.2882 |
|  | XP\_065053058\_5367-model40.1 | -14.9368 |
|  | XP\_065053058\_5367-model40.2 | -14.7802 |
| # cluster 41 - Size: 1 |  |  |
|  | XP\_065053058\_5367-model41 | -16.2281 |
| # cluster 42 - Size: 1 |  |  |
|  | XP\_065053058\_5367-model42 | -16.1838 |
| # cluster 43 - Size: 1 |  |  |
|  | XP\_065053058\_5367-model43 | -16.131 |
| # cluster 44 - Size: 1 |  |  |
|  | XP\_065053058\_5367-model44 | -16.0688 |
| # cluster 45 - Size: 1 |  |  |
|  | XP\_065053058\_5367-model45 | -15.9968 |
| # cluster 46 - Size: 1 |  |  |
|  | XP\_065053058\_5367-model46 | -15.9468 |
| # cluster 47 - Size: 1 |  |  |
|  | XP\_065053058\_5367-model47 | -15.5384 |
| # cluster 48 - Size: 1 |  |  |
|  | XP\_065053058\_5367-model48 | -15.4644 |
| # cluster 49 - Size: 1 |  |  |
|  | XP\_065053058\_5367-model49 | -15.4464 |
| # cluster 50 - Size: 1 |  |  |
|  | XP\_065053058\_5367-model50 | -15.2759 |
| # cluster 51 - Size: 1 |  |  |
|  | XP\_065053058\_5367-model51 | -15.2397 |
| # cluster 52 - Size: 1 |  |  |
|  | XP\_065053058\_5367-model52 | -15.2231 |
| # cluster 53 - Size: 1 |  |  |
|  | XP\_065053058\_5367-model53 | -14.3832 |
| # cluster 54 - Size: 1 |  |  |
|  | XP\_065053058\_5367-model54 | -14.2448 |
| # cluster 55 - Size: 1 |  |  |
|  | XP\_065053058\_5367-model55 | -14.0263 |
|  |  |  |
